# Using Different Low-Profile Abutments for Assisting Mandibular Implant Overdenture: A Split-Mouth Study

**DOI:** 10.1155/2022/8738220

**Published:** 2022-04-09

**Authors:** Ahmed K. Khalifa, Mohammad Mahmoud Alokda, Mohamed Moustafa Said

**Affiliations:** ^1^Department of Prosthodontics, Galala University, Suez, Egypt; ^2^Department of Prosthodontics, Mansoura University, Mansoura, Egypt

## Abstract

**Background:**

Using a pair of different low-profile abutments to assist mandibular implant overdenture (MIOD) in limited restorative space is questionable due to the different morphology.

**Objective:**

To investigate the marginal bone level (MBL) change and peri-implant-tissue health (PITH) around a pair of OT Equator^®^ and Locator^®^ suprastructures assisting MIOD.

**Methods:**

Seventeen edentulous patients received MIOD assisted by OT Equator^®^ and Locator^®^. MBL change was investigated at the implant loading (T1), after six months (T2) and twelve months (T3) of implant loading. PITH was evaluated at T2 and T3.

**Results:**

There was within abutment significant difference in MBL change after T2 and T3 of loading for Locator (0.05 ± 0.02 and 0.32 ± 0.08, respectively) (*P*=0.01); and for Equator (0.11 ± 0.08 and 0.21 ± 0.09, respectively) (*P*=0.01). Also, there was a significant difference between Locator and Equator on the modified plaque index (MPI) after T3 (*P*=0.01). The significant results were recorded for the MPI at T2 and T3 for Locator (0.92 ± 0.26 and 1.5 ± 0.51, respectively) (*P*=0.01) and for Equator (0.82 ± 0.26 and 1.42 ± 0.51, respectively) (*P*=0.003). For modified bleeding index, there was significant difference at T2 and T3 for Locator (0.57 ± 0.19 and 1.14 ± 0.41, respectively) (*P*=0.03) and for Equator (0.46 ± 0.22 and 1.07 ± 0.41, respectively) (*P*=0.01). For gingival index, there was significant difference at T2 and T3 for Locator (0.57 ± 0.11 and 1.28 ± 0.35, respectively) (*P*=0.001) and for Equator (0.35 ± 0.21 and 1.1 ± 0.46, respectively) (*P*=0.001).

**Conclusions:**

Using different pairs of the low-profile OT Equator^®^ and Locator^®^ abutments to assist MIOD is clinically acceptable based on the MBL change and PITH outcomes.

## 1. Introduction

The longevity of implants is basically dependent on implant-bone integration. Initial breakdown of the implant-tissue integration mainly begins at the marginal alveolar bone. This is multifactorial behavior graded from systemic [1] to biomechanical [2] factors. Mandibular implant overdenture (MIOD) assisted by two anterior implants is rapidly becoming the first choice in treatment planning for edentulous mandibular ridge [3, 4]. This leads to the advent of overdenture anchorage systems that evolve in design to meet the demand of both patients and clinicians. Attachment systems used for relating overdenture to implants include nonsplinting attachments (magnets, resilient studs, and telescopic crowns) and splinting systems as (bar-clip construction with different bar shapes). The choice of attachment system is based on patient's demand, cost, and planned load distribution to the implants and surrounding structure. The solitary anchoring system can be beneficial in the limited prosthetic space and self-alignment but with less resistance to lateral forces and limited forces distribution [5]. While the use of splinting anchorage system enhances the biomechanics of MIOD, it requires sufficient restorative space and special construction technique.

The use of a low-profile attachment, like Locator^®^ and OT Equator^®^, may develop more useful alternative when prosthetic space is compromised and stress distribution required enhancing serviceability of implants [6]. Locator^®^ system is a self-aligning double retention cylinder which consists of an implant screw-metallic and metallic cap lined with nylon replacements with different colors according to their degree of retention. This attachment has been widely used due to its retention capacity, low maintenance, and tolerance of angulations between implants [7]. OT Equator^®^ system was introduced in 2007 to offer a new option of low-profile attachments [8]. The vertical height and diameter of OT Equator^®^ are 2.1 and 4.4 mm, respectively. The nylon caps are highly flexible and allow self-alignment without decrease in retention. But, the system has not been widely researched for its clinical efficacy. Also, there is no investigation for the suitability of using different pairs of the low-profile attachment to assist MIOD to broad the treatment plan options at complicated clinical situations such as limited available restorative space.

To ensure the serviceability of MIOD, monitoring of the marginal bone level (MBL) changes around the neck of implant through the treatment and follow-up can be done successively by periapical X-ray film [9]. However, some clinical indices [10–12] for peri-implant-tissue health (PITH) elaborate a dominant idea about the implant-tissue integration.

At conducting the clinical trial, the standardization of cases is an illusion due to variation among cases. Other obstacles are the number of involved patients in each compared group which, in turn, reflects on the cost of the research work and the period of follow-up. In split-mouth study, one side is considered the control for the other. Besides, split-mouth study expunges the bias at randomization or inclusion criteria and gives clear results without cofounders based on patients' conditions [13].

The purpose of this split-mouth study is to investigate the (MBL) change and PITH around a pair of OT Equator^®^ and Locator^®^ abutments assisting MIOD. The null hypotheses are that there is no difference between both anchor systems and can be used instantly to treat edentulous patients.

## 2. Materials and Methods

Seventeen completely edentulous patients were selected for this study. According to medical history, the included patients were free from diseases affecting the rate of bone resorption, tissue health, and/or ability of patients to fulfill planned follow-up period. Patients with history of TMJ disorders, clenching, bruxism, and/or smoking were excluded. All patients were with suitable interarch space to receive the planned MIOD and Angel's class I maxillomandibular relationship verified by tentative jaw relation. The elapsed time since last tooth extraction was at least six months. All patients had sufficient bone quantity and quality in the mandibular intraforaminal region verified by cone beam CT for placement of required implant without bone augmentation. The procedures of research and follow-up were explained for patients for written approval according to the regulation of ethical committee number (16050618) by the Faculty of Dentistry, Mansoura University. For each patient, the following of presurgical, surgical, pick-up, and follow up and evaluation procedures were done.

After primary and secondary maxillomandibular impression making and jaw relation transfer to semiadjustable articulator by face-bow record, the artificial teeth (Acrostone, Egypt) were set according to lingualized occlusion scheme [14]. After clinical try in, the denture was flasked, finished, and polished in conventional manner. Patients received denture and were followed-up for one month to verify denture occlusion and adaptation.

**Figure 1:**
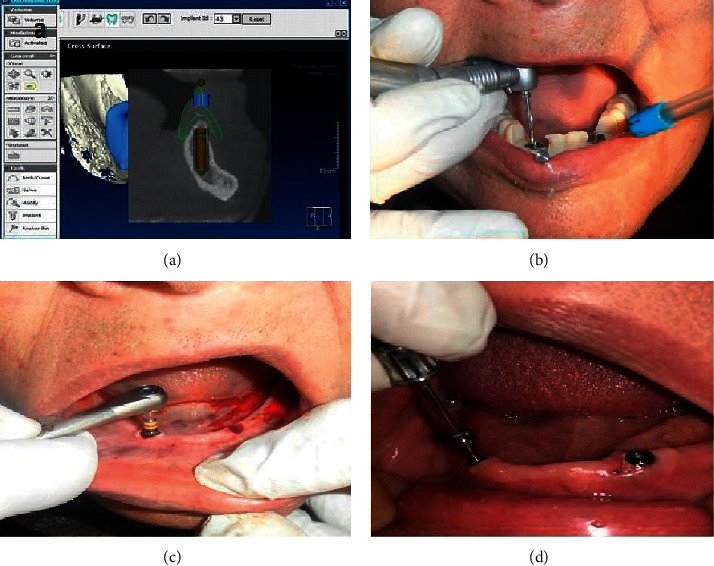
Surgical procedures for fixture mounting; (a) virtual planning (sagittal view); (b) drilling sequence through mounted surgical guide; (c) fixture mounting; (d) screwing of healing abutments.

The mandibular complete denture was duplicated [15]. The duplicated denture was used during double scanning by CBCT with modification of the intaglio by adding gutta-percha opposing to two canine areas [16]. Images were loaded into 3D image planning software (In2guide software by Cybermed) to design position and angulation of implants virtually [17] (Figure 1(a)). A mucosal supported stereolithographic surgical guide with two metal sleeves and anchor pins was printed according to implant planned sites (Figure 1(b)).

A dose of antibiotic (Augmentin, GSK, UK) prophylaxis was administered 1 hour preoperatively. Under local anesthesia (lignocaine 2%, Alex Co., Egypt), Universal Surgical Kit (In2Guide Universal Kit, Cybermed Inc) was used to perform full sequence drilling through the anchored guide (Figure 1(b)). Each patient received two implants (3.7 × 11.5 mm; Neo Biotech, Seoul, South Korea.) (Figure 1(c)). The intaglio of the denture opposite to implants was recessed and filled with soft liner (Promedica, Germany). The patient was instructed for soft diet and home care with frequent recall and follow-up. After three months, healing abutments were mounted for two weeks with required modification of the intaglio of mandibular denture (Figure 1(d)).

**Figure 2:**
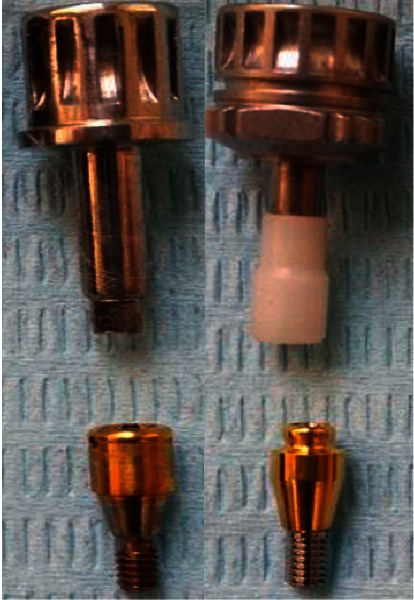
OT Equator (right) and Locator (left) abutments with mounting drivers.

**Figure 3:**
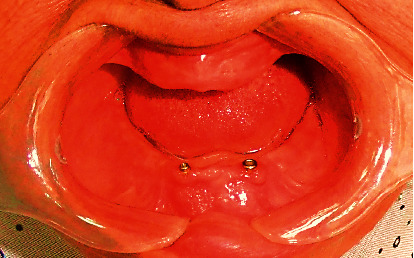
OT Equator (left) and Locator (right) abutments mounted intraorally.

OT Equator^®^ (Rhein 83, Bologna, Italy) and Locator^®^ abutments (Kerator System, New York, USA) were mounted to internal hex of the left and right implant, respectively, using the special mounting key for each abutment (Figure 2). Translucent ring and white spacer ring were placed on the head of each OT Equator^®^ and Locator^®^, respectively. Metal caps with clear and pink inserts for Equator^®^ and Locator^®^, respectively, were placed on abutments and the denture was modified to include abutments with caps without rocking (Figure 3).

The pick-up was done by adding auto-polymerized acrylic resin (Acrostone, Egypt) in modified intaglio of the denture and under patient's occlusion. After complete polymerization, excess released from preprepared lingual vents and the fitting surface was trimmed. The patient was instructed for home care and regular follow-up.

**Figure 4:**
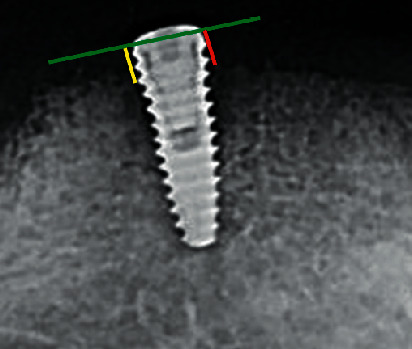
Radiographic tracing for periapical X-ray film. The mesial and the distal lines (red and yellow, respectively) parallel for each other and perpendicular to reference (green) line.

The evaluation was done for MBL, PITH, according to the planned study design. A digital periapical radiograph was performed immediately after implant insertion (T1) in the visit of implant loading, after 6 months (T2) and after 12 months of implant loading (T3). The capture was done according to the long cone paralleling technique with custom jig [18]. Radiographic measurements were traced on the radiograph by using software (Corel Draw v12, Corel Co., Canada) at 30x. The distance from traced reference line representing implant neck to the highest point of the marginal alveolar bone indicates aggregation of bone around the implant was measured and rounded to the nearest 0.01 mm (Figure 4). MBL change was computed by subtracting the corresponding bone levels before loading period from bone level after a year from insertion. MBL was measured, mesial and distal aspects, and subsequently averaged to determine the mean MBL change [19, 20]. Measurements of modified plaque index (MPI) [10], modified bleeding index (MBI) [11] and gingival index (GI) [12] for both implants were assessed at two observational periods, after six months of implant loading and one year after loading.

For sample size calculation, preliminary analysis for two cases (not involved in final screening) was done before the study. We obtained 0.055 ± 0.08 mean difference and standard deviation MBL between both anchor systems. Assuming the dependency of observations at 80% power of study, an alpha error of 5% for one-tail test, the sample size was calculated by *G*^*∗*^ power program (Kiel University, Germany) [21]. The estimated sample size was 15 cases, and two cases were added to compensate the drop down of participants (if any). After follow-up period, data were tabulated and statistically analyzed by Statistical Package for Social Sciences program (SPSS), version 23 (SPSS IBM Inc., England). Quantitative data were described as mean and standard deviation (SD) after testing normality with Shapiro-Wilk test. Student's and repeated *t*-tests were conducted to check between-subject and within-subject differences at *P* value ≤ 0.05 significant level.

## 3. Results

There was a significant difference in MBL change for the time period T1 to T2 and T2 to T3 for Locator^®^ (0.05 ± 0.02 and 0.32 ± 0.08, respectively) (*P*=0.01) and for OT Equator^®^ in the same time intervals (0.11 ± 0.08 and 0.21 ± 0.09, respectively) (*P*=0.01) (Table 1). Also, there was a significant difference between the Locator^®^ and OT Equator^®^ on the MPI after 12 months (*P*=0.01). The significant results were recorded for the MPI at 6 and 12 months (0.92 ± 0.26 and 1.5 ± 0.51, respectively) around Locator^®^(*P*=0.001) and in the same time periods for OT Equator^®^ (0.82 ± 0.26 and 1.42 ± 0.51, respectively) (*P*=0.003). For GI, there was significant difference at 6 and 12 months (0.57 ± 0.11 and 1.28 ± 0.35, respectively) around Locator^®^(*P*=0.001) and in the same time periods for OT Equator^®^ (0.35 ± 0.21 and 1.1 ± 0.46, respectively) (*P*=0.001). For MBI, there was significant difference at 6 and 12 months (0.57 ± 0.19 and 1.14 ± 0.41, respectively) around Locator^®^(*P*=0.03) and in the same time periods for OT Equator^®^ (0.46 ± 0.22 and 1.07 ± 0.41, respectively) (*P*=0.01) (Table 2).

## 4. Discussion

This study evaluated the effect of Locator^®^ and OT Equator^®^ on the peri-implant hard and soft tissue changes. As a unit of the low-profile attachments category, OT Equator^®^ had the minimal number of research investigations [8, 22]. While magnet is one of low-profile attachments, it was not involved in the study due to the different modes of action which is based on magnetic forces [23] rather than frictional contact in Locator^®^ [24] and OT Equator^®^ [8].

In this study, MIOD was assisted by combined anchorage from Locator^®^ and OT Equator^®^. Split mouth study design was carried out previously to study implant behavior by applying different system in each quadrant [25]. The combined assessment by Locator^®^ and Equator® was designed based on the low-profile nature of both systems [26]. While both attachments are comparable in height [27], the variation of stud attachment height is tolerable within a limited range [28, 29]. Based on biomechanical analysis, stress pattern of the low-profile attachment is favorable comparing to other form of attachment systems as the minimal height of these attachments permits adequate thickness of overlaying acrylic resin which results in a greater compressive stresses neutralizing the occlusal load [30]. The action of nylon inserts for both attachments is proportionate [8]. The self-alignment action is common for both systems [31]. Adding to that, the variety of nylon inserts with different retentive capacity for both attachments permits the control of the retentive forces offered by both attachment systems.

Regarding the MPI, there was a statistically significant difference within attachment at six and twelve months. With advance of time, and due to the resiliency of the Locator^®^ and OT Equator^®^, minute food debris accumulates to the intaglio of MIOD [32]. This movement is exaggerated with OT Equator^®^ due to loss of precision fit of nylon insert by wearing [26]. Adding to that, the day and night wearing of the overdenture after substitution of postinsertion complains. This was mentioned by Kuoppala et al. as 91% of overdenture wearers keep their dentures at night [33]. This can also be attributed to the improper home care and oral hygiene measures by elder patients [34]. This disagrees with Ammar et al. who studied MIOD with OT Equator^®^ showing no significant increase of MPI [32], while the significant increase for MPI with Locator^®^ after 12 months could be explained by the larger diameter comparing to Equator^®^ which allows more debris accumulation circumferentially.

The results revealed significant increase of MBI and GI within time. This may be correlated to MPI [35]. The gingival inflammation is statistically dependent with plaque score [36]. Thus, increasing plaque accumulation increases roughness around abutment which enhances bacterial adhesion and launching inflammation sequalae [37, 38]. Another reason is the posterior movement of the denture beneath the anterior two implants. Anterior-implant design allows the anterior implants to act as a fulcrum for posterior denture display which enhances gingival enlargement around abutments [39]. Translucent and white spacer rings were placed on the head of each OT Equator^®^ and Locator^®^, respectively, to block out the area surrounding the abutment creating space to allow rotation of metal denture cap over the attachment providing resiliency and range of movement of caps above the abutment. This blocked space acts as plaque reservoir and enhances bacterial accumulation [32]. Beside that the matrix housing movement against the peri-implant mucosa during mastication flares the gingival and bleeding indices [37].

While there was a significant difference with MPI with OT Equator^®^, there was insignificant difference in MBL for both attachments. This independency between MBL and PITH is in agreement with Visser et al. [39] after monitoring 180 implants which assisted two different designs of overdenture. Obviously, the scores of PITH indices do not, in most of the time, predict MBL change [40]. Other explanation is the short period of follow-up (12 months). Monitoring MBL change for one year after implant loading is considered an indicator for the biomechanical adaptation to the precipitated load [41–43]. According to our result, the annual bone loss after loading is within the range of previous study [44]. The significant difference of MBL within each implant by time can be related to the early bone modeling as suggested by Cehreli et al. [44]. Bone loss after implant loading is still a considerable unavoidable outcome [45]. However, the result is in the acceptance range (about 0.4 after loading) according to Galindo-Moreno et al. [46]. In another study, there was insignificant marginal bone loss within the first six months after using OT Equator® with MIOD [32]. John et al. [47] concluded that the small diameter attachment plays a role to minimize the stresses to marginal bone. On the other hand, the insignificant difference of the MBL for both attachments may be due to the low-profile design for both. This was in line with Abdelhamid et al. [4] who illustrated that low-profile design has a role in dissipating occlusal loads through the abutment to the marginal bone around implant. Although both attachment caps shared similar occlusal load, it seemed that the available thickness of the acrylic overdenture overlying the low-profile design attachments acted as a mechanical absorber for the applied load decreasing the induced stresses on the marginal bone [48]. According to previous study [49], the abutment height is the main cofactor in MBL. The cantilever action of the low-profile abutment assists the suppression of MBL. This also combined by Vervaeke et al. [50] after monitoring 39 cases. The vertical and rotational resiliency provided by nylon insert for both attachments minimizes the stress transfer to the supporting tissues. However, this was found to conflict with the findings by El-Anwar et al. [51] and Celik and Uludag [52] who noticed greater stress values at using Locator® attachments. The double matrix-patrix relationship in the Locator® attachment has been mentioned as a reason for such stresses. This could explain the insignificant increased MBL change with Locator® by the result of this research.

The number of the participants was calculated based on the effect size and the planned power of the study, while further investigations are required with larger sample size to overcome the considerable limitations. Also, the short time of follow-up (12 months) is considered as a limitation of this study to discover the rate of MBL change.

## 5. Conclusion

Within the limitation of the study, the result suggested that the low-profile Locator^®^ and OT Equator^®^ are comparable in the MBL change and PITH. The MBL change for both suprastructures is in the acceptable range within the first year of implant loading. The companion between different two low-profile suprastructures to assist MIOD is acceptable from the perspective of implant-tissue interaction. Further researches are required to approve this overdenture design.

### 5.1. Limitation: The Limited Member of Cases and Follow-Up Period

### 5.2. Recommendation

Due to the intimate morphological form of both Locator^®^ and OT Equator^®^, it is recommended to be used simultaneously to assist MIOD.

## Figures and Tables

**Table 1:** MBL change within 12 months of implants loading.

	T1 to T2		T2 to T3	
	Mean ± SD^*∗*^	*P* ^*∗∗*^	Mean ± SD	*P* ^*∗∗*^
Locator	0.05 ± 0.02	<0.01	0.32 ± 0.08	0.01
OT Equator	0.11 ± 0.08	<0.01	0.21 ± 0.09	0.01
*P* ^*∗*^	0.6		0.2	

^*∗*^Mean and standard deviation in millimeters. ^*∗*^Student's *t*-test. ^*∗∗*^Paired *t*-test.

**Table 2:** Peri-implant-tissue change with both attachments after 6 and 12 months of implant loading.^*∗*^

	Locator		OT Equator		*P* value
	6 m	12 m	6 m	12 m	
	*Mean SD*	*Mean SD*	*Mean SD*	*Mean SD*	
MPI	0.92 0.26^*a*^	1.50 0.51^*a,c*^	0.82 0.26^*b*^	1.42 0.51^*b,c*^	0.001^*a*^ 0.003^*b*^ 0.01^*c*^
MBI	0.57 0.19^*a*^	1.14 0.41^*a*^	0.47 0.22^*b*^	1.07 0.41^*b*^	0.03^*a*^ 0.01^*b*^
GI	0.57 0.11^*a*^	1.28 0.35^*a*^	0.35 0.21^*b*^	1.1 0.46^*b*^	0.001^*a*^ 0.001^*b*^

^*∗*^Similar letters represent statistically significant difference in the same row. MPI, modified plaque index. MBI, modified bleeding index. GI, gingival index.

## Data Availability

Because the data are a part of a big project (mentioned in the ethical committee approval letter attached in the supplement files), so the authors cannot make it freely available in this stage. The authors of this article with another authors (third-party) are planning for consequent publications based on comparing data with another overdenture design. We can offer data after finishing the project completely.

## Additional Points

This study was conducted in accordance with all the provisions of the local human subjects' oversight committee guidelines and policies (the name of institute is removed temporary due to blind reviewing). The approval code for this study is 16050618.
